# Spontaneously Immortalised Nonhuman Primate Müller Glia Cell Lines as Source to Explore Retinal Reprogramming Mechanisms for Cell Therapies

**DOI:** 10.1002/jcp.31482

**Published:** 2024-11-28

**Authors:** Ahmed Salman, Arantxa Bolinches‐Amorós, Tina Storm, Daniela Moralli, Paulina Bryika, Angela J. Russell, Stephen G. Davies, Alun R. Barnard, Robert E. MacLaren

**Affiliations:** ^1^ Nuffield Department of Clinical Neurosciences University of Oxford Oxford Oxfordshire UK; ^2^ Welcome Centre for Human Genetics University of Oxford Oxford Oxfordshire UK; ^3^ Department of Chemistry University of Oxford Oxford Oxfordshire UK; ^4^ Department of Pharmacology University of Oxford Oxford Oxfordshire UK; ^5^ Oxford Eye Hospital Oxford Oxfordshire UK

**Keywords:** cellular therapy, differentiation, glia, nonhuman primate model, reprogramming, retina, self‐renewal

## Abstract

Cell replacement therapies for ocular diseases characterised by photoreceptors degeneration are challenging due to poor primary cell survival in culture. A stable retinal cell source to replace lost photoreceptors holds promise. Müller glia cells play a pivotal role in retinal homoeostasis by providing metabolic and structural support to retinal neurons, preventing aberrant photoreceptors migration, and facilitating safe glutamate uptake. In fish and amphibians, injured retinas regenerate due to Müller‐like glial stem cells, a phenomenon absent in the mammalian retina for unknown reasons. Research on Müller cells has been complex due to difficulties in obtaining pure cell population and their rapid de‐differentiation in culture. While various Müller glia cell lines from human and rats are described, no nonhuman primate Müller glia cell line is currently available. Here, we report spontaneously immortalised Müller glia cell lines derived from macaque neural retinas that respond to growth factors and expand indefinitely in culture. They exhibit Müller cells morphology, such as an elongated shape and cytoplasmic projections, express Müller glia markers (VIMENTIN, GLUTAMINE SYNTHASE, glutamate‐aspartate transporter, and CD44), and express stem cell markers such as PAX6 and SOX2. In the presence of factors that induce photoreceptor differentiation, these cells show a shift in gene expression patterns suggesting a state of de‐differentiation, a phenomenon known in reprogrammed mammalian Müller cells. The concept of self‐renewing retina might seem unfeasible, but not unprecedented. While vertebrate Müller glia have a regeneration potential absent in mammals, understanding the mechanisms behind reprogramming of Müller glia in mammals could unlock their potential for treating retinal degenerative diseases.

## 1 Introduction

Humans are unique in their reliance on sight as a dominant sense and sight loss has a significant impact, on both individuals and societies. Loss of photoreceptors, the light sensitive cells in the retina at the back of the eye, represents one of the main causes of retinal visual impairment and blindness. Cell replacement therapies of the photoreceptors are challenging due to the poor survival of those cells in culture. Current therapeutic approaches to restore vision, including prosthetic devices and cell replacement and gene therapies, involve introducing a foreign object into the eye (Pearson et al. 2012; Wirth, Parker, and Ylä‐Herttuala 2013; Karamali et al. 2022). A source of progenitor cells within the retina to replace lost cells would certainly be an ideal therapeutic approach. Müller glia, astrocyte‐like radial cells, are the major glial cells in the retina (Goldman 2014), they originate from multipotent progenitor cells that give rise to multiple retinal neurons (Turner and Cepko 1987). They maintain retinal homoeostasis and integrity and contribute to overall retinal structure and function such as; transport of molecules between different cells in the retina, supporting neurons by releasing trophic factors, releasing neurotransmitters and controlling ionic balance. Müller glia furthermore contribute to the outer segment assembly and recycling of retinal chromophore for light transduction (Bringmann et al. 2009; Reichenbach and Bringmann 2013). In response to retinal injury, Müller glia generate a response referred to as ‘reactive gliosis’ which involves changes in cell morphology, physiology, and biochemistry as part of a global response to damage in the retina. The principle of retinal regeneration in fish and amphibians has been documented for several decades now (Raymond and Hitchcock 1997), with Müller glia believed to be the source of retinal regeneration. In Teleost fish such as zebrafish, Müller glia, in the injured retina, show a regeneration potential involving reprogramming and proliferation of retinal stem cells to regenerate all major cell types of the retina (Lindsey and Powers 2007; Mensinger and Powers 1999; Sherpa et al. 2008). Although there are studies that indicated Müller glia may regenerate the retina in mammals such as in rat and chicken (Fischer and Reh 2001; Acad Sci USA 2004), evidence that Müller glia can regenerate the retina in mammals is weak (Ooto 1996). Müller glia can proliferate into stem cell progenitors in vitro and when supplemented with growth factors that induce differentiation, they can acquire neural morphology and express postmitotic retinal neuronal markers (Lawrence 2007a; Angbohang et al. 2016). However, Müller glia are unable to regenerate the mammalian retina in vivo, the mechanisms of why they are unknown. But, it is believed that factors induce retinal degeneration in humans might prevent a permissive environment for Müller glia to proliferate into retinal progenitors, and hence differentiate into retinal neurons (Bringmann et al. 2006). Many retinal degenerative conditions that lead to blindness are associated with abnormal Müller glia proliferation, which ultimately leads to failure to repair the retina (Bringmann et al. 2009). To our knowledge, no spontaneously immortalised Müller glia cell line derived from a Nonhuman primate neural retina currently exist, which can represent a useful in vitro model for understanding the mechanisms of Müller glia regenerative potential in the mammalian retina. in addition, sourcing a Nonhuman primate tissue is easier than the human tissue. Here, we report isolation, immortalisation, and characterisation of three primary cell lines (cell line 1, cell line 2 and cell line 4), derived from the neural retinas of rhesus macaque monkeys. These cell lines expanded indefinitely in culture without the addition of growth factors, express Müller glia specific markers including VIMENTIN, GLUTAMINE SYNTHASE, glutamate‐aspartate transporter (GLAST) and CD44 as shown by immunocytochemistry (ICC) and reverse transcription PCR. They also express stem cells markers such as paired‐box protein PAX6 and SRY (sex determining region Y)‐box 2 (SOX2). The cell lines can be frozen and thawed from different passages without losing their characteristics. Since immortalisation is a characteristic of stem cells, we examined the Nonhuman primate cells for expression of stem cell progenitors, and whether they can differentiate into retinal neurons under conditions known to stimulate neural differentiation. These cell lines represent a flexible, accessible, and rapidly expandible source of clinically relevant cells. They provide a valuable resource for exploring the fundamentals of Müller glia reprogramming and potential cell therapies for retinal degenerative diseases.

## 2 Methods

### 2.1 Isolation and Immortalisation of Primary Müller Glia Cells From Adult Macaque Neural Retina

Neural retinas of four young adult rhesus macaques (between 4 and 16‐year‐old) obtained from Porton Down Facility, Wiltshire UK, were harvested and processed individually by enzymatic dissociation using Papain Dissociation System (Worthington, USA) according to the manufacturer's instructions. Briefly, after removal of cornea and lens by holding the optic nerve with a pair of forceps, four radial cuts through the sclera were made to flatten the eye cap. The vitreous was gently dislodged using closed pair of forceps. Then, using a punch cutter (1 mm diameter) a section of the retina attached to vitreous, RPE and choroid from the posterior side of the eye cap was cut to avoid disturbing the ciliary body. The neuroretina was then gently dissected away from those fragments and immediately rinsed in PBS, cut in small pieces and carefully separated from vitreous traces before being transferred to a 15 mL falcon tube containing 700 µL of papain/DNase solution for papain dissociation (Worthington Biochemical Corporation). The sample suspension was incubated for 45 min at 37°C shaking the tube every 5 min followed by gentle trituration with pipette. To stop the papain dissociation 500 µL of EBSS were added to the tube and the mixture was centrifuged for 5 min at 300 g. The cell pellet was resuspended in 450 µL EBSS + 50 µL ovomucoid inhibitor + 25 µL DNAse and layered on top of 600 ul of ovomucoid solution. After forming a discontinuous density gradient, the sample was centrifuged for 6 min at 70 g. The pellet containing dissociated retinal cells was resuspended in fresh warm Neurobasal‐A medium containing 2% B27, 1% N2% and 1% G5 supplements, 0.8 mM l‐glutamine, 100 units/mL penicillin and 100 µg/mL streptomycin (all reagents from Gibco). 50,000–100,000 cells/cm^2^ were seeded on poly‐d‐lysine/laminin‐coated plates and incubated at 37°C and 5% CO_2_ for 24 h to allow the cells to attach. Medium was changed after 24 h to remove nonattached neuronal and dead cells, after which it was replaced every 3–4 days until a homogeneous population was obtained. Once flasks reach full confluency, cells were detached with TryPLE Express (Gibco) and subsequently split to obtain subcultures maintained on DMEM/F‐12 medium supplemented with GlutaMAX, 10% FBS, 100 units/mL penicillin and 100 µg/mL streptomycin (Gibco) at 37°C and 5% CO_2_. The number of cell division per passage is estimated according to the rate of growth until cells reach confluency for subculturing, which is approximately five divisions per passage. Cells were examined for characteristic morphology and expression of Müller glia specific markers VIMENTIN, GLUTAMINE SYNTHASE, GLAST and CD44 as judged by ICC staining or reverse transcription PCR (RT‐PCT) of cell lysate.

### 2.2 Confocal Analysis of Müller Glia and Other Retinal Neuronal Markers by ICC

20,000 cells/cm^2^ were seeded on poly‐d‐lysine/laminin‐coated coverslips (Scientific Laboratory Supplies; 354068) and incubated at 37°C and 5% CO_2_. Once reached 80% confluency, cells were rinsed with PBS (Gibco) and fixed with 4% methanol‐free formaldehyde (Pierce, Thermo Scientific) for 15 min at room temperature. To prevent autofluorescence, fixed cells were permeabilised in 150 mM Glycine for 15 min at room temperature, then blocked with 10% Goat Serum + 5% BSA + 0.5% Triton X‐100 for 90 min. Cells were incubated overnight at 4C with primary antibodies diluted in PBS + 10% Goat Serum + 5% BSA, these included rabbit monoclonal anti‐VIMENTIN (1:1000; Abcam ab92542), rabbit polyclonal anti‐GLUTAMINE SYNTHASE (1:100; Abcam ab228590), rabbit polyclonal anti‐glutamate‐aspartate transporter (GLAST) (1:100; Abcam ab416), rat monoclonal anti‐CD44 (1:100; Abcam ab119348), rabbit monoclonal anti‐PAX6 (1:500; Abcam 195045), rabbit polyclonal anti‐SOX2 (1:100; Abcam 97959), mouse monoclonal anti‐RHODOPSIN (1:500; Abcam ab98887), rat monoclonal anti‐CD73 (1:200; BD‐Biosciences BD550738). Rabbit Ig isotype matching those of the test antibodies (1:500; Abcam ab208569). After washing with PBS + 0.1% Tween 20, cells were incubated with appropriate secondary antibodies diluted in PBS for 1 h at room temperature protected from light henceforth, these included goat anti‐rabbit AF555 (1:500; Thermo‐Fisher Scientific A21428), goat anti‐mouse AF647 (1:500; Thermo‐Fisher Scientific A32728) and goat anti‐rat AF647 (1:500; Thermo‐Fisher Scientific A21247). Cell nuclei were counterstained with Hoechst 33342 (Invitrogen, Thermo Scientific) diluted 1:1000 in PBS for 5 min. After washing with PBS, the coverslips were mounted on slides with ProLong Diamond Antifade Mountant (Thermo‐Fisher Scientific; P36965). Cells stained with secondary antibody only (anti‐rabbit IgG 555 made in goat, anti‐mouse IgG 647 made in goat and anti‐rabbit IgG 555 isotype control made in goat) as negative controls. Fluorescent imaged were captured using a confocal microscope (LSM 710; Carl Zeiss, Germany) operating in multitrack mode for FITC, DAPI and red fluorochromes.

### 2.3 mRNA Expression of Müller Glia and Other Retinal Neuronal Markers by RT‐PCR

Following detachment of cell monolayers, total RNA was extracted from cell lysates using the RNeasy kit (Qiagen) according to the manufacturer's instructions. 1 µg of total RNA was reverse‐transcribed using the QuantiTech Reverse Transcription Kit (Qiagen) according to manufacturer's instructions. The expression of specific Müller glia and retinal genes (Table 1) was assessed by RT‐PCR using 50 ng of cDNA as template. The PCR reaction was performed with Immomix (Bioline) with the following conditions: enzyme activation at 95°C for 10 min, denaturation at 95°C for 15 s, annealing 55°C–60°C for 30 s, extension 72°C for 45 s (×35 cycles) and a final extension 72°C for 8 min. *β‐ACTIN* expression was used as a control housekeeping gene. Thereafter, PCR products were analysed on 2% agarose gels.

**Table 1:** RT PCR primers.

Name	Primer
*CALRETININ* ‐Fw	CCTGGAGATTGTGCTCTGCA
*CALRETININ* ‐Rv	TCCGTGTGTGTGATCCACTG
*GLUL* ‐Fw	AGTTTCCACCACAAGGGCAA
*GLUL* ‐Rv	TTTCGGTGACAGAAGCCCTC
*CRALBP* ‐Fw*	TATTCCCCATCCCCCACCTT
*CRALBP* ‐RV*	GCCTCTCCTCAACTGTCCTG
*NOTCH1* ‐Fw	TGAGATGTGTGGACTGTGGC
*NOTCH1* ‐Rv	TCCTACAAAACACGGGAGCC
*NR2E3* ‐Fw	CAGCAGAGATGCCCACTGAA
*NR2E3* ‐Rv	TGCCACCCAGAAGTAACCAC
*OPN1* LW ‐Fw	TTGAAGGCCCCAACTACCAC
*OPN1* LW ‐Rv	GAAGCCTTCCAGGACACACA
*OPN1* SW ‐Fw	TTCCTGCCAAAGCTGTAGCA
*OPN1* SW ‐Rv	AGTGATGGCAGTTTGGGGAG
*PAX6* ‐Fw	AGTGAATCAGCTCGGTGGTG
*PAX6* ‐Rv	CCGCTGTGAGCTAGCTCTAC
*RECOVERIN* ‐Fw	AGTGAATCAGCTCGGTGGTG
*RECOVERIN* ‐Rv	CCGCTGTGAGCTAGCTCTAC
*RHODOPSIN* ‐Fw	CAGGTGACTTCCAGAGGCTG
*RHODOPSIN* ‐Rv	GACCCTCTTAGCAGCAGCAA
*TUBB3* ‐Fw	ACATGAACGACCTGGTGTCC
*TUBB3* ‐Rv	AAAGCAGGGTACAGTGTCGG
*NEURO FILAMENT 160KD* ‐Fw	GTGAACCACGAGAAGGCTCA
*NEURO FILAMENT 160KD* ‐Rv	ATGGCCTCCTTGTTCTGCTC

### 2.4 Karyotype Analysis of Nonhuman Primate Müller Glia Cells

Karyotyping analysis performed for cell line 1 (34), cell line 2 (36) and cell line 4 (39). Metaphase spreads were harvested following synchronisation with nocodazole (Merck; M1404) 0.1 mg/mL. The cells were swollen in buffered hypotonic solution (Genial Helix; GGS‐JL006) and fixed in Carnoy's fixative. For chromosome counting, slides were mounted in VECTASHIELD Antifade Mounting Medium with DAPI (2‐B Scientific; H‐1200‐10). M‐FISH was performed with the 24 × Cyte probe‐set (Metasystem Probes; D‐0425‐060DI) following to the manufacturer's instructions. To distinguish between chromosomes 12 and 13, the following BAC probes were used: RP11‐50E1 (mapping to MMA 13p and HSA 2p25); RP11‐67K11 (MMA 13q and HSA 2p14); RP11‐ 157E8 (MMA 12p, and HSA 2q31); RP11‐15L18 (MMA 12q and HAS 2q37). The BACs were labelled by nick translation using a commercial kit, (Abbott Molecular; 07J000) following the manufacturer instructions to incorporate either ChromaTide Alexa Fluor 594‐5‐dUTP or ChromaTide Alexa Fluor 488‐5‐dUTP (ThermoFisherer Scientific; C11400 and C11397, respectively). The probes were hybridised using a standard protocol (Rayner et al. 2019). All cytogenetics preparations were analysed using the Leica Cytovision software, on an Olympus BX‐51 epifluorescence microscope equipped with a JAI CVM4+ progressive‐scan 24 fps B&W fluorescence CCD camera.

### 2.5 Differentiation of Nonhuman Primate Müller Glia Cells Into Neural Phenotype

The spontaneously immortalised Nonhuman primate Müller cells from cell lines 1, 2 and 4, were maintained as a monolayer in culture in DMEM F‐12 supplemented with l‐GlutaMAX, 10% FBS and 100 μ/mL Penicillin‐Streptomycin as described previously (Lawrence 2007a; Limb et al. 2002). For passaging, cells were detached by incubation with TrypLE Express Enzyme (Thermo‐Fisher Scientific; 12605036) and seeded at a 1:6 dilution of the original flask. Cells were cultured at 37°C with 5% CO_2_ and medium replenishment on average twice per week. For differentiation studies, cells were seeded at a density of 800 cells per cm^2^ on Matrigel with the presence or absence of fibroblast growth factor 2 (FGF2) (Sigma‐Aldrich) at a final concentration of 20 ng/mL, Taurine) (Sigma‐Aldrich) at a final concentration of 20 μM, retinoic acid (Sigma‐Aldrich) at a final concentration of 5 μM, and insulin‐like growth factor 1 (IGF‐1) (ReproTech) at a final concentration of 100 ng/m. Medium and growth factors were replenished every 2 days. Cells were examined by ICC and RT‐PCR to assess expression of retinal stem cells and postmitotic neuronal markers. Following 7 days in culture, ICC assessment for the expression of retinal stem cell markers was performed using the following primary antibodies; rabbit monoclonal anti‐PAX6 (1:500; Abcam 195045), rabbit polyclonal anti‐SOX2 (1:100; Abcam 97959). Expression of postmitotic retinal neurons was examined by ICC using the following primary antibodies; mouse monoclonal anti‐NEUROFILAMENT 160 kDa (Merch Millipore; MAB5254), chicken polyclonal anti‐ßIII TUBULIN (Abcam, ab9354), mouse monoclonal anti‐RHODOPSIN (1:500; Abcam ab98887), and mouse monoclonal anti‐RECOVERIN (1:500; Abcam [6A55CD6] ab31928). Expression of Müller glia markers was examined by ICC with rabbit polyclonal anti‐GLUTAMINE SYNTHASE (1:100; Abcam ab228590). RT‐PCR analysis was performed using primers detecting the following retinal stem cells and post mitotic neuronal markers (Table 1).

## 3 Results

### 3.1 Establishment and Characterisation of the Spontaneously Immortalised Müller Glia Cells From Nonhuman Primate Neural Retina

Nonhuman primate primary Müller glia cells were cultured following a previously published protocol for human Müller glia cells (Limb et al. 2002). The primary cultures of four cell lines were obtained from retinas that were isolated via a punch made in the inner retina through the sclera and away from the ciliary body. This was done to avoid contamination of the cultures with putative progenitor cells that have previously been described to reside at the ciliary margin (Ahmad, Tang, and Pham 2000; Tropepe et al. 2000). The NHP Müller glia cell lines were expanded in culture in the absence of EGF, except the freshly dissociated retinal cells which were grown in the presence of B27, N2 and G5 supplements to support the primary culture of retinal neural and glial cells in serum free media. The cells were examined for Müller glia characteristics at the time of the first passage and subsequently after 20 and 30 passages (approximately 100‐150 divisions). Cell lines that continued dividing after 30 passages were considered immortalised (Shay, Wright, and Werbin 1991; Irfan Maqsood et al. 2013). A Müller glia cell line derived from human subjects were considered immortalised if continued division after 20 passages (Lawrence 2007a). In this study we chose passage number 30 as the minimum passage number for immortalised cell lines to add certainty and robustness to our choice of immortalised cell lines. Out of four primary derived cultures, three cell lines underwent more than 30 passages; cell line 1 underwent 32 passages (approximately 160 divisions), cell line 2 underwent 38 passages (approximately 190 divisions) and cell line 4 underwent 53 passages (approximately 265 divisions). Those cell lines were cryopreserved and thawed several times without losing their characteristics such as maintaining a spindle‐like appearance and showing cytoplasmic projections. All cell lines (1, 2, and 4) exhibited the same characteristics at different passages.

**Figure 1:**
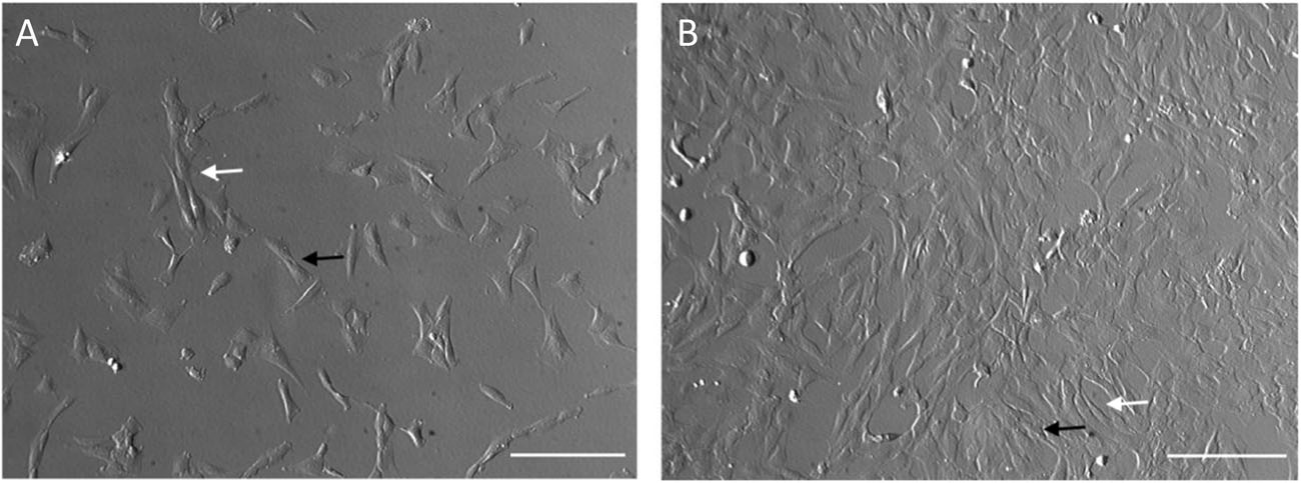
Morphological features of the Müller glia cell lines. (A) Sub‐confluent monolayer of nonhuman primate glia cells showing cytoplasmic projections and bipolar shape appearance. (B) Confluent monolayer of cells showing spindle‐like appearance which was a characteristic of these cells in culture.

Morphological features of spindle‐like Müller glia were evident in the phase‐contrast micrographs of the nonhuman primate primary cells. Non confluent monolayer of cells (Figure 1A) spread evenly in the culture plate, adopt a bipolar morphology, forming cytoplasmic projections and displaying rough membrane appearance. When confluent (Figure 1B), they acquired an elongated shape and displayed a fibroblast‐like shape, However, they retained their rough membrane appearance.

### 3.2 Gene Expression Patterns of Müller Glia Cell Lines

**Figure 2:**
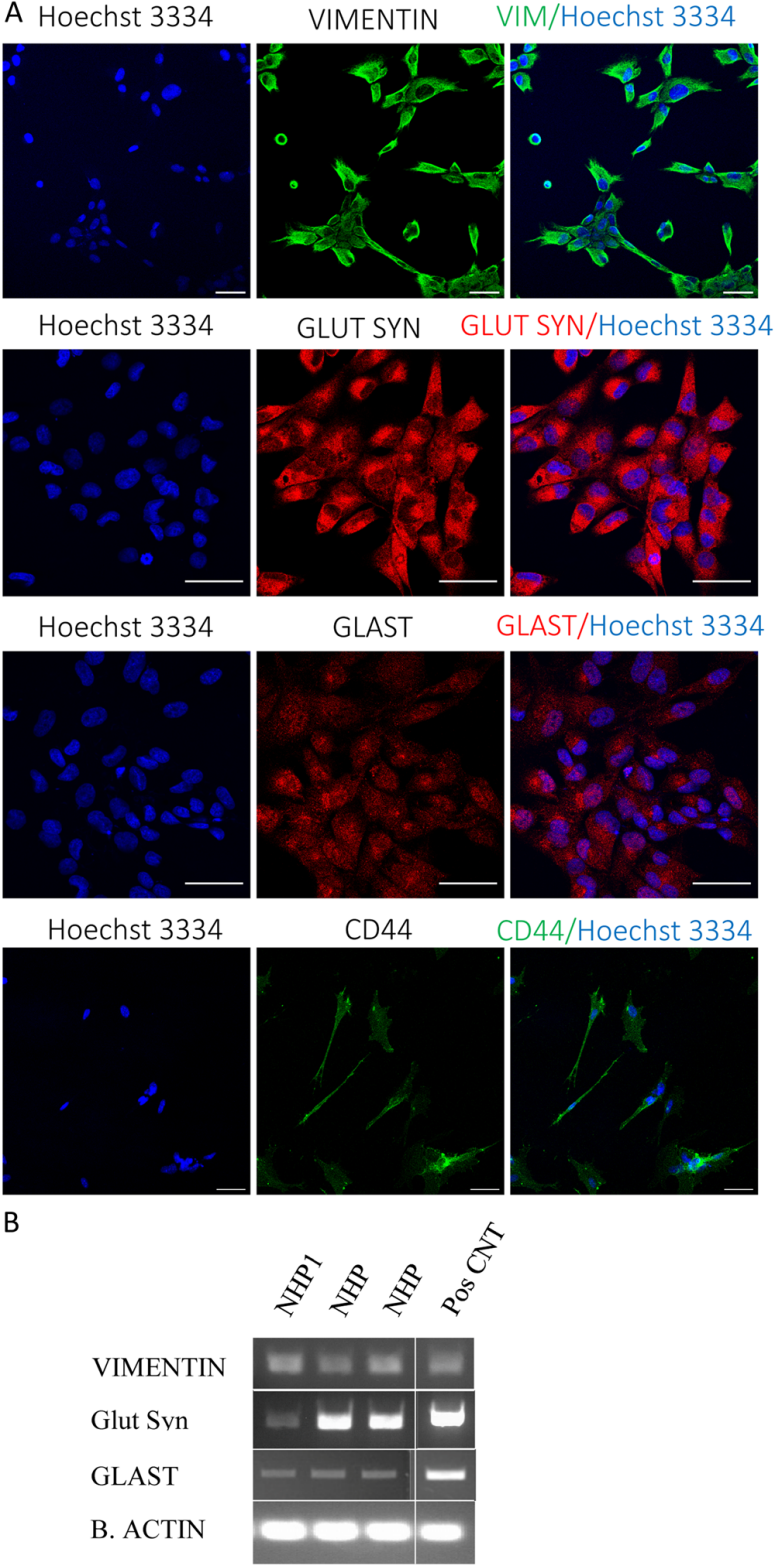
Confirmation of Müller glia phenotypes in spontaneously immortalised cells derived from adult rhesus macaque neural retina. (A) Confocal images of fixed cells subjected to immunofluorescence staining against Müller glia specific markers (VIMENTIN (VIM), GLUTAMINE SYNTHASE (Glut Syn), glutamate‐aspartate‐transporter (GLAST) (red) and CD44 (FITC). Cells stained with secondary antibody only as controls (anti‐rabbit IgG 555 made in goat, anti‐rat IgG 647 made in goat and anti‐rabbit IgG 555 isotype control made in goat), see Figure S3. Hoechst 33342(blue) was used as a counterstain. Scale bar = 50 mm unless otherwise indicated. (B) Reverse transcription‐polymerase reaction (RT‐PCR) products showing expression of Müller glia specific markers (VIMENTIN (*VIM*), glutamine synthase (*GLUL*) and *GLAST*) by the different nonhuman primate (NHP) cell lines. Primary cells from Rhesus macaque neural retinal culture were used as a positive control to assess the fidelity of PCR primers. The house‐keeping gene *b‐ACTIN* used as a positive control.

Müller glia cells characteristics were identified by examining gene expression patterns of well‐known Müller glia specific markers by ICC and reverse transcription‐polymerase chain reaction (RT‐PCR), the latter used as an end point analysis of binary (present/absent) answer of gene expression. Immunofluorescence staining and RT‐PCR analysis of the primary Nonhuman primate cells lines, cultured in the absence of extracellular matrix and without the addition of growth factors, cell line 1, 2 and 4 showed they express well‐known Müller glia specific markers including VIMENTIN, GLUTAMINE SYNTHASE, glutamate‐aspartate transporter (GLAST) and CD44 (Figure 2A,B). Expression of VIMENTIN, GLUTAMINE SYNTHASE was also evident with RT‐PCR analysis (Figure 2B), expression of GLAST was not seen with RT‐PCR. Only a small proportion of cells expressed glial fibrillary acidic protein (GFAP), particularly during early passages of primary cultures (data not shown). This result was not unexpected since not all mammalian Muller glia cells express GFAP especially if grown under controlled cell culture conditions (Lewis et al. 1994). Additional controls included the omission of the primary antibodies and the use of isotype control from the same species in which most of the primary antibodies raised, specifically rabbit IgG, (Figure S3).

### 3.3 Gene Expression Patterns of Retinal Stem Cells and Postmitotic Neurons Markers in the Different Müller Glia Cell Lines

**Figure 3:**
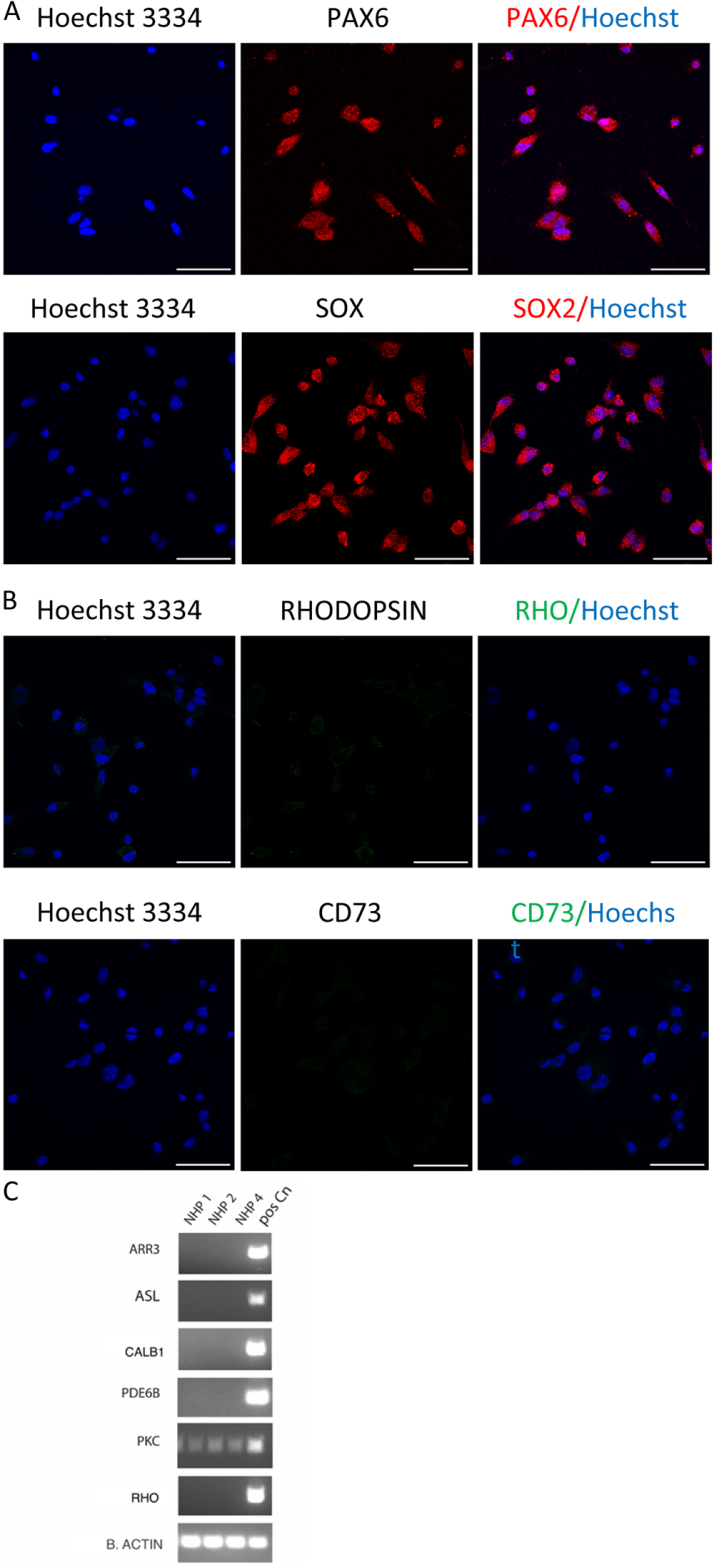
Gene expression analysis of markers of neural stem cells and postmitotic retinal neurons in spontaneously immortalised nonhuman primate cells lines. (A) Confocal images of fixed cells subjected to immunofluorescence staining against stem cell specific markers (PAX6 and SOX2) (red). (B) Immunofluorescence staining against postmitotic neuronal markers (RHODOPSIN and CD73) ((FITC). Cells stained with secondary antibody only as controls. (anti‐rabbit IgG 555 made in goat, anti‐mouse IgG 647 made in goat and anti‐rabbit IgG 555 isotype control made in goat) see Figure S4. Hoechst 33342 (blue) was used as a counterstain. Scale bar = 50 mm unless otherwise indicated. (C) Reverse transcription‐polymerase reaction (RT‐PCR) products showing expression of postmitotic retinal neurons (retinal cone arrestin‐3 (*ARR3*), ISL Lim homeobox 1 (*ISL‐1*), calbindin 1 (*CALB1*), phosphodiesterase 6b (*PDE6B*), protein kinase C alpha (*PKC*), rhodopsin (*RHO*). Primary cells from Rhesus macaque neural retinal culture were used as a positive control to assess the fidelity of PCR primers. The house‐keeping gene *b‐ACTIN* used as a positive control.

Investigation of expression of neural stem cell markers was performed by ICC of cells cultured in the absence of extracellular matrix and without the addition of growth factors. Immunofluorescent staining confirmed that these cells express stem cell specific markers including paired box‐6 (PAX6) and SRY (sex determinant region)−2 (SOX2) (Figure 3A). Despite some degree of perinuclear/cytoplasmic staining for both markers which could be due to antibody specificity, but the staining is primarily nuclear as expected. In contrast, ICC analysis of the same cells revealed lack of expression of different postmitotic retinal neurons including RHODOPSIN (RHO) and CD73, identifying rod photoreceptors, (Figure 3B). Similarly, RT‐PCR of mRNA extracted from the different cell lines revealed lack of expression of several postmitotic retinal neuronal markers including (i) retinal cone arrestin‐3 (*ARR3*) identifying cone photoreceptors (Craft, Whitmore, and Wiechmann 1994); (ii) ISL Lim homeobox 1 (*ISL‐1*), identifying ganglion cells (Bejarano‐Escobar et al. 2015); (iii) calbindin 1 (*CALB1*), identifying horizontal cells (Mitchell et al. 1995) and (iv) phosphodiesterase 6b (*PDE6B*) and rhodopsin (*RHO*), identifying rod photoreceptors (McLaughlin et al. 1993) (Figure 3C). However, expression of protein kinase C alpha (*PKC*), a marker of bipolar cells (Nakashima 2002), was observed. Additional controls included the omission of the primary antibodies and the use of isotype control from the same species in which most of the primary antibodies raised, specifically rabbit IgG, (Figure S4).

### 3.4 Karyotypic Features of the Nonhuman Primate Müller Glia Cells

**Figure 4:**
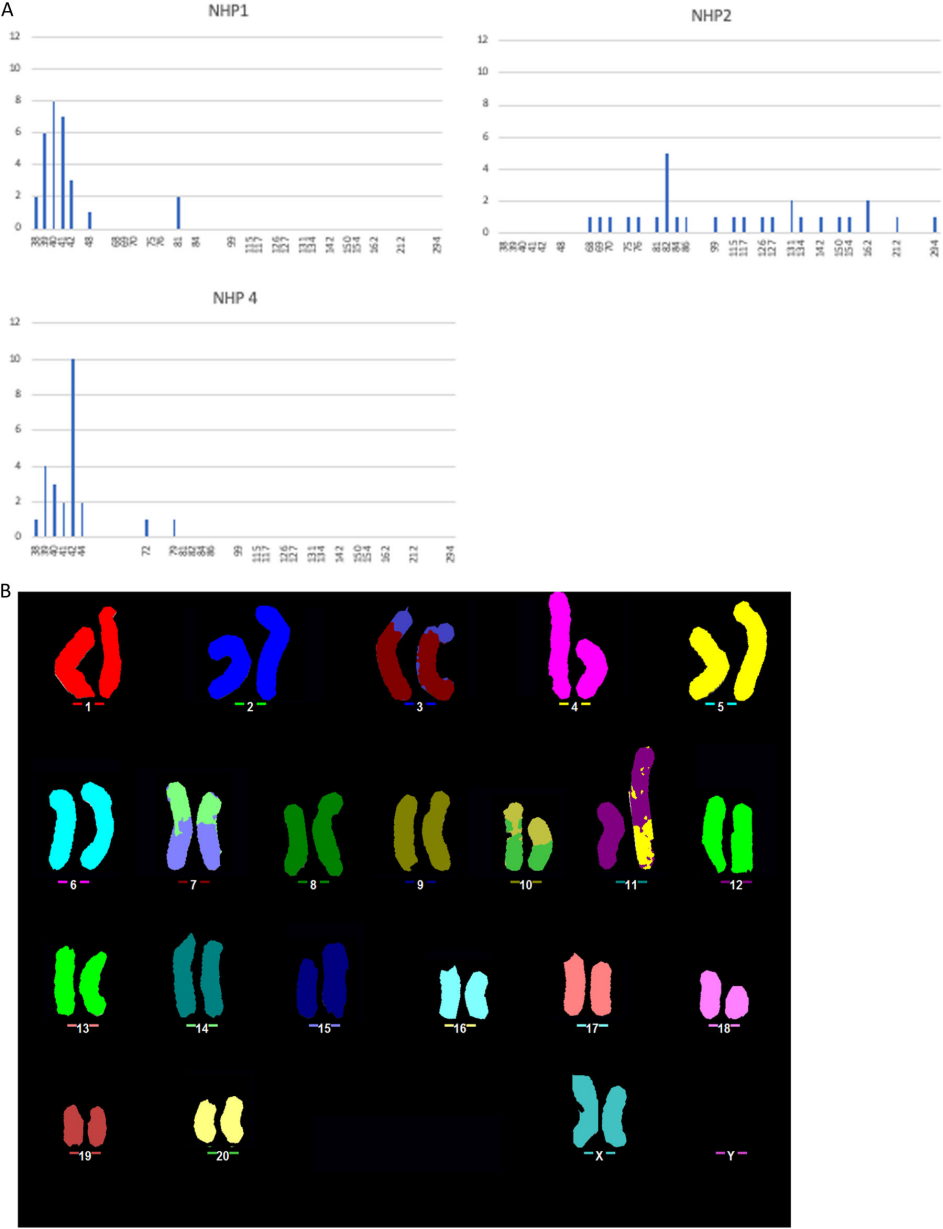
Karyotyping analysis of nonhuman primate Müller glia cell lines (A) Chromosome number distribution of the different NHP cell lines. (B) MFISH analysis of cell line 4 showing normal number of rhesus macaque chromosomes of *n* = 42.

The chromosome complement of the three cell lines was analysed firstly by chromosome counting of DAPI stained metaphases. The rhesus macaque monkey has 42 chromosomes (Soares et al. 1982). Karyotyping analysis of cell lines 1, 2 and 4 (Figure 4) indicates that while cell line 1 and 2 presented abnormal karyotype, cell line 4 has a normal modal number of *n* = 42 (Figure 4A and Table 2). The *Macaca mulatta* and human karyotype are syntenic, and it is standard procedure to analyse the karyotype of the rhesus monkey using human probes (Sangpakdee et al. 2018). Thus, the chromosomes of cell line 4 were further characterised by M‐FISH with a human probe‐set. This experiment showed that the cell line has karyotype 42, XX, t(11;5) (Figure 4B). The animal from which the cells were sourced was male, but we did not identify any Y material. This led us to conclude that, along with the translocation between chromosome 11 and 5, the chromosome Y was lost, and an extra chromosome X was gained, possibly during the spontaneous immortalisation. *Macaca mulatta* autosome (MMA) 12 and 13 are both syntenic to *Homo Sapiens* autosome (HSA) 2. Since the M‐FISH probe‐set used cannot distinguish between the MMA 12 and 13, a further FISH experiment was carried out with human probes mapping to the p and q arm of these two chromosomes. The FISH showed that cell line 4 has two normal copies of chromosome 12 and 13 (see Figures S1 and S2).

**Table 2:** Cell counting index for karyotyping analysis of spontaneously immortalised nonhuman primate Müller glia cell lines.

NHP1		NHP2		NHP4	
Chromosome number	Number of cells	Chromosome number	Number of cells	Chromosome number	Number of cells
38	2	38		38	1
39	6	39		39	4
40	8	40		40	3
41	7	41		41	2
42	3	42		42	10
				44	2
48	1	48			
68		68	1		
69		69	1		
70		70	1		
				72	1
75		75	1		
76		76	1		
				79	1
81	2	81	1	81	
		82	5	82	
84		84	1	84	
		86	1	86	
99		99	1	99	
115		115	1	115	
117		117	1	117	
126		126	1	126	
127		127	1	127	
131		131	2	131	
134		134	1	134	
142		142	1	142	
150		150	1	150	
154		154	1	154	
162		162	2	162	
212		212	1	212	
294		294	1	294	

### 3.5 Evidence of Differentiation Potential of the Spontaneously Immortalised NHP Müller Glia Cells

**Figure 5:**
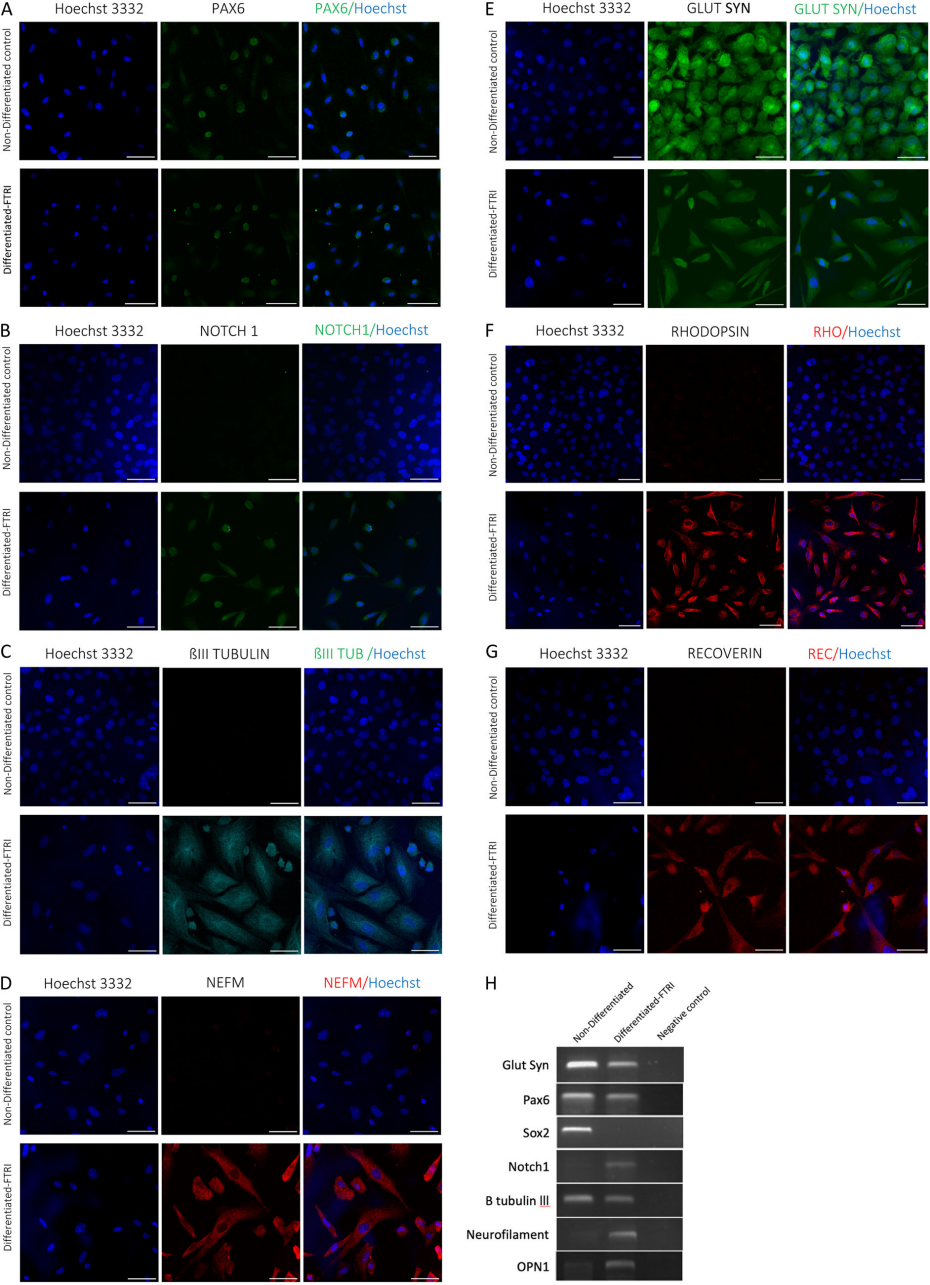
Differentiation of Müller progenitor cells following short‐term culture w/w FGF2, Taurine, Retinoic acid, IGF1 (FTRI) differentiation complex. Only positive cells were shown to illustrate the pattern of gene expression before and after differentiation. Confocal images of fixed cells subjected to immunofluorescence staining against neural stem cell specific markers PAX6, NOTCH1, and ßIII TUBULIN (A, B, and C respectively), against the postmitotic neuronal marker Neurofilament (NEFM) (D), against the Müller glia specific marker GLUTAMINE SYNTHASE (GLUT SYN) (E), and against the photoreceptors specific markers RHODOPSIN (RHO) and RECOVERIN (REC) (F and G, respectively). Cells stained with secondary antibody only as controls. (anti‐rabbit IgG 555 made in goat, anti‐mouse IgG 647 made in goat and anti‐rabbit IgG 555 isotype control made in goat) see supplementary Figure 4. Hoechst 33342 (blue) was used as a counterstain. Scale bar = 50 mm unless otherwise indicated. (H) Reverse transcription‐polymerase reaction (RT‐PCR) products showing expression of postmitotic retinal neurons glutamine synthase (*GLUL*), *PAX6, SOX2, NOTCH1*, ßIII TUBULIN *(TUBB3)*, 160 kDa Neurofilament (*NEF3*) and short wavelength Opsin (*OPN1*).

Results from the literature show that adult human Müller glia with stem cell characteristics express photoreceptors specific markers upon culture with fibroblast growth factor type‐2 (FGF2), taurine, retinoic acid and insulin‐like growth factor type‐1 (IGF‐1) (FTRI) (Jayaram et al. 2014). To induce differentiation of the spontaneously immortalised NHP Müller cells we used conditions that are known to promote neural differentiation (Coles et al. 2004; Kelley, Turner, and Reh 1995; Klassen et al. 2004; Lawrence 2007b; Yang 2002) in the presence of FTRI differentiation mix. Since cell line 4 showed normal karyotype of *n* = 42, it was chosen for the subsequent differentiation protocol described below. Cells cultured with a low density (800 cells per cm^2^) on Matrigel with the presence or absence of FTRI were examined for expression of retinal stem cell markers and postmitotic retinal neurons after 7 days in culture by ICC and RT‐PCR. Cell morphology changed after 3–7 days in culture including loss of obvious bipolar shape and reduction in density (Figure S5). To exclude the possibility that the cells express early differentiation markers before FTRI treatment, expression levels of early differentiation markers such as homeobox transcription factor (*OTX2)*, neural retina leucine zipper (*NRL*) and cone‐rod homeobox (*CRX*) were measured by RT PCR and no expression of any of the markers was observed (Figure S6A). Following 7 days in culture, we observed expression of retinal stem cell markers with ICC including PAX6, the master regulator of early retinal development (Marquardt et al. 2001) in the presence and absence of FTRI (Figure 5A); NOTCH1, an important signalling pathway at several stages of retinal development including the differentiation of retinal ganglion cells and Müller glia (Ahmad, Zagouras, and Artavanistsakonas 1995; Silva, Ercole, and McLoon 2003) in the presence of FTRI only (Figure 5B); and ßIII TUBULIN, immature neurons marker (Meyer et al. 2004; Osborne and Larsen 1996), in the presence of FTRI (Figure 5C). These results were further confirmed by RT‐PCR (Figure 5H). Staining for 160 kDa neurofilament (NEFM), a marker of ganglion, amacrine and horizontal cells (Gutierrez, McNally, and Canto‐Soler 2011), was observed in cells cultured with FTRI only, staining of NEFM was not observed in the absence of FTRI in both ICC and RT‐PCR (Figure 5D,H, respectively). Expression of the Müller glia marker glutamine synthetase was observed in the presence or absence of FTRI (Figure 4E). The level of expression was remarkably reduced in the presence of FTRI, whereas in the absence of FTRI, higher levels of expression were observed as expected. This was evident in the RT PCR (Figure 4H), however, endpoint reverse transcription PCR analysis is semi‐quantitative and used here as a binary answer indicating presence/absence of markers, so a precaution needs to be considered when interpreting the levels of RT‐PCR product in this case. Expression of photoreceptors specific markers RHODOPSIN and RECOVERIN, markers of rod photoreceptors (Seiler and Aramant 1994), has also been observed in the presence of FTRI and was absent in the undifferentiated controls (Figure 5F,G, respectively). Expression of the cone specific marker short‐wavelength opsin (OPN1‐SW) (Calkins 2001) was observed with RT PCR analysis (Figure 5H). Cell type specific markers such as photoreceptors were also examined revealing presence of some and absence of other markers (Figure S6B).

## 4 Discussion

Permanent sight loss and blindness often result from cell death within the retina, predominantly photoreceptor death, which leaves the remaining neural visual pathway intact. The idea of the self‐renewal potential of cells within the retina is therapeutically appealing. The regeneration potential of Müller glia is well documented in the literature (for a recent review, see [Salman, McClements, and MacLaren 2021]). However, whilst the mammalian Müller glia can acquire stem cell characteristics, which leads to retinal repair (Powell et al. 2013; Ramachandran, Fausett, and Goldman 2015; Fausett and Goldman 2006; Qin, Barthel, and Raymond 2009; Nagashima, Barthel, and Raymond 2013; Kassen et al. 2007), their regeneration potential is limited, a phenomenon that is not fully understood to date. Further investigation of the molecular mechanisms of this in the mammalian Müller glia is required to unlock the puzzle, but suitable mammalian cell models are required.

Spontaneously immortalised human Müller glia cell lines have been reported in the literature in the past (Limb et al. 2002; Lawrence 2007b), which helped broaden our understanding of the mechanisms of Müller cells in the mammalian system. However, human primary cell lines are difficult to obtain and establish due to the substantial challenges obtaining suitable tissue. Nonhuman primate represents an ideal substitute to the human counterpart, but no established spontaneously immortalised Müller glia cell line has been characterised so far. The present study demonstrates spontaneously immortalised Müller glia cell lines isolated from rhesus macaque monkeys, grown under standard culture conditions, responded to epidermal growth factors, and could be expanded indefinitely without the presence of growth factors. The present findings that cells isolated from macaque monkeys become spontaneously immortalised in vitro and exhibit both Müller glia and stem cell characteristics, similar to their human counterpart, suggest that these cells can be used as a suitable cell culture model to study mammalian Müller glia regeneration potential. The established cell lines show morphological characteristics of Müller cells in culture such as spindle‐like appearance and one of which, cell line 4, is the most stable cell line with a normal karyotype of 48 chromosomes.

The spontaneously immortalised NHP Müller cell lines not only express mRNA for well known markers of Müller glia (as judged by RT PCR), but also express proteins (as demonstrated by immunostaining) that are characteristically expressed by these cells such as VIMANTIN, GLUTAMINE SYNTHASE, GLAST and CD44 (Limb et al. 2002; Das et al. 1992; Eastlake et al. 2019; Linser and Moscona 1979; Pow and Barnett 2000; Sarthy et al. 1998). End point analysis PCR is used here as a binary (present/absent) indication of mRNA transcripts of the target genes used. Which represent a semi‐quantitative measure to indicate presence/absence of markers. Other Müller glia markers such as CRALBP and EGF‐R although have been shown to be expressed in Müller glia in the literature, however, these markers were also reported as markers of other retinal cells such as RPE (CRALPB, [Kennedy et al. 2003]) and astrocytes (CRALBP [Johnson et al. 1997] and EGF‐R, [Liu et al. 2006]), hence have been excluded from the study.

The present study reports the expression profile of the Müller glia cells throughout the immortalisation process at the gene expression level, represented by ICC and RT‐PCR. Other reports in the literature describe a shift in the proteomics profiling of mammalian immortalised Müller glia cells upon adaptation in culture, represented by altered levels of proteins involved in Müller cells physiological functions, such as downregulation or absence of proteins involved in glycolysis, transmitter recycling, CO_2_ syphoning, visual pigment cycle (Hauck, Suppmann, and Ueffing 2003). In contrast, cytoskeletal proteins, as well as proteins involved in motility proliferations and retinal injury, such as GFAP, which has been reported to be upregulated in response to retinal injury (Bignami and Dahl 1979; Wen et al. 1995), were shown to be upregulated. In the present study, we noted upregulation in GFAP expression during initial Müller glia culture isolation (passage 0), which rapidly downregulated during subsequent passages (passages 1 and 2). We explain this due to the initial stress exerted on the cells during the isolation process, once the cells settle in culture, that stress is no longer present, hence levels of GFAP was downregulated afterwards, and continues throughout the immortalisation process.

It is important to emphasise that the cell lines in this study were grown as a monolayer of adherent cells from the neural retina without growth factors. This contrasts with other studies where retinal stem cells were derived from the ciliary body and cultured as sphere colonies. Despite being originally derived from the neural retina, the spontaneously immortalised cells show both Müller glia and retinal stem cell characteristics judged by the presence of stem cell markers such as PAX6 and SOX2. In addition, we did not observe expression of other postmitotic retinal neuronal markers such as RHODOPSIN and CD73 by ICC, nor the following markers by RT‐PCR; retinal cone arrestin‐3 (*ARR3*), ISL Lim homeobox 1 (*ISL‐1*) (ganglion cell marker), calbindin 1 (*CALB1*) (horizontal cell marker), and phosphodiesterase 6b (PDE6B) and rhodopsin (*RHO*) (photoreceptors markers) throughout the immortalisation period, which further supports their Müller glia phenotype. We attribute the presence of bands of protein kinase C alpha (*PKC*) to unspecific PCR primers.

The spontaneously immortalised cells, devoid of growth factors during immortalisation, demonstrated a lack of differentiation induction during this period. However, when exposed to an extracellular matrix (Matrigel) and growth factors like fibroblast growth factor type‐2 (FGF2), taurine, retinoic acid, and insulin‐like growth factor type‐1 (IGF‐1) known to induce neural stem cell differentiation (FTRI) (Jayaram et al. 2014), the cells exhibited a shift in expression patterns of various neural and retinal postmitotic markers, indicating a state of de‐differentiation. This phenomenon aligns with known characteristics of mammalian Müller stem cells (see (Salman, McClements, and MacLaren 2021) for further details). The NHP Müller glia cells described herein retain expression of the retinal stem cell marker PAX6 in the presence of FTRI, but also express NOTCH1. Expression of this marker further indicates their neural stem cell potential because Notch1 signalling is not only important for Müller glia differentiation, but also for other retinal cell type like retinal ganglion cells. The de‐differentiation potential of the cells is further demonstrated by the expression of immature neuronal markers such as ßIII TUBULIN and 160 kDa neurofilament (NEFM), the latter also a marker of ganglion, amacrine and horizontal cells (Gutierrez, McNally, and Canto‐Soler 2011), consistent with undergoing a process of de‐differentiation.

The NHP Müller glia cells we describe retain expression of Müller glia markers such as glutamine synthetase in the presence and absence of FTRI. The presence of FTRI also induced expression of other postmitotic retinal markers such as RHODOPSIN and RECOVERIN (photoreceptors specific markers, [Seiler and Aramant 1994]), which was not observed in the absence of FTRI. However, it is a little premature to assume these cells are differentiating into photoreceptors yet without further functional analysis such as grafting and electrophysiology, expression of photoreceptor markers such as RORA and RORB and absence of others such as OTX, NRL and NR2E3 for example, further strengthen the assumption that the cells are undergoing a state of dedifferentiation rather that committing to a specific fate. The reduction in cell density after FTRI induction suggest a halt in proliferation, which is a feature of cells undergoing dedifferentiation. Nonetheless, the fact that the spontaneously immortalised NHP Müller cells are showing a shift in the gene expression patterns upon stimulation with factors that induce differentiation is promising to say the least.

Many questions remain about the regeneration potential of mammalian Müller glia before their therapeutic use in cell replacement therapies can be explored. However, the ability to derive, expand, and culture mammalian cells with Müller glia and stem cell characteristics indefinitely, cryopreserve them for extended periods without losing phenotypic characteristics, and induce differentiation in culture suggests that once unknown factors inhibiting Müller glia regeneration in mammals are identified, these cells may hold promise for clinical application in the near future.

## Author Contributions

**Ahmed Salman:** conception and design, collection and/or assembly of data, manuscript writing. **Arantxa Bolinches‐Amorós:** conception and design, manuscript revision. **Tina Storm:** manuscript revision. **Daniela Moralli:** collection and/or assembly of data. **Paulina Bryika:** collection and/or assembly of data. **Angela J. Russell:** supervision, manuscript revision. **Stephen G. Davies:** supervision, manuscript revision. **Alun R. Barnard:** conception and design, supervision, manuscript revision. **Robert E. MacLaren:** conception and design, supervision, manuscript revision.

## Conflicts of Interest

The authors declare no conflicts of interest.

## Supporting information

Supporting information.

## Data Availability

The data that support the findings of this study are available from the corresponding author upon reasonable request.
