# Supplementary material for: Spontaneously Immortalised Nonhuman Primate Müller Glia Cell Lines as Source to Explore Retinal Reprogramming Mechanisms for Cell Therapies
Source: J Cell Physiol. 2024 Nov 28;240(1):e31482. doi: 10.1002/jcp.31482 (PMC11774137; doi:10.1002/jcp.31482)
Supplement: Supplementary file 1 — Supporting information. [file JCP-240-0-s001.docx]

**Spontaneously immortalised Non-Human Primate Müller glia cell lines as source to explore retinal reprogramming mechanisms for cell therapies**

Ahmed Salman^1^, Arantxa Bolinches Amoros^1,3^, Tina Storm^1^, Daniela Moralli^4^, Paulina Bryika^4^, Angela J. Russell^2,3^, Stephen G. Davies^2^, Alun R. Barnard^1^, Robert E. MacLaren^1,5^*

^1^Nuffield Department of Clinical Neurosciences, University of Oxford, Oxford, UK.

^2^Department of Chemistry, University of Oxford, Oxford, UK

^3^Department of Pharmacology, University of Oxford, Oxford, UK

^4^Welcome Centre for Human Genetics, University of Oxford, Oxford, UK

^5^Oxford Eye Hospital, Oxford, UK

*Corresponding author


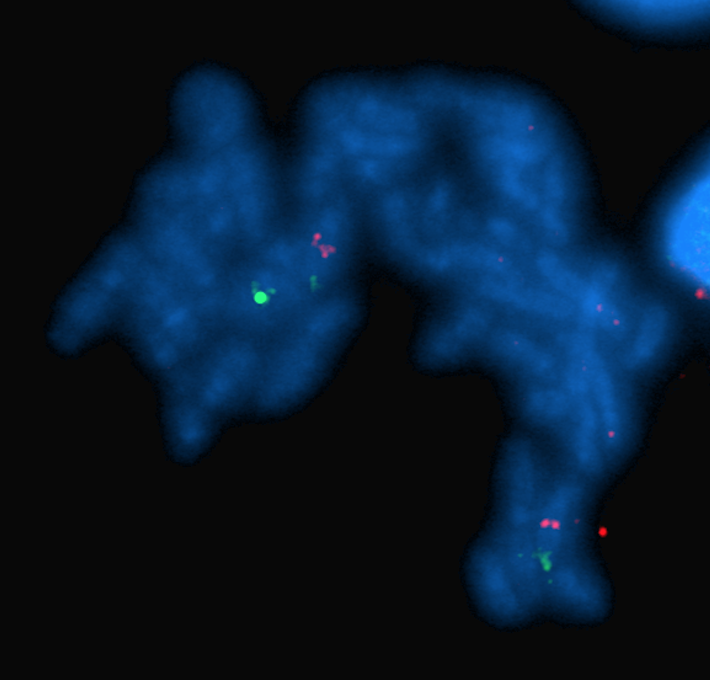


**Supplementary Figure 1.** NHP4- Image showing probes localised to macaque chromosome 12 (yellow arrows) with red signals obtained on the p arm and green signals on the q arm of chromosome 12.


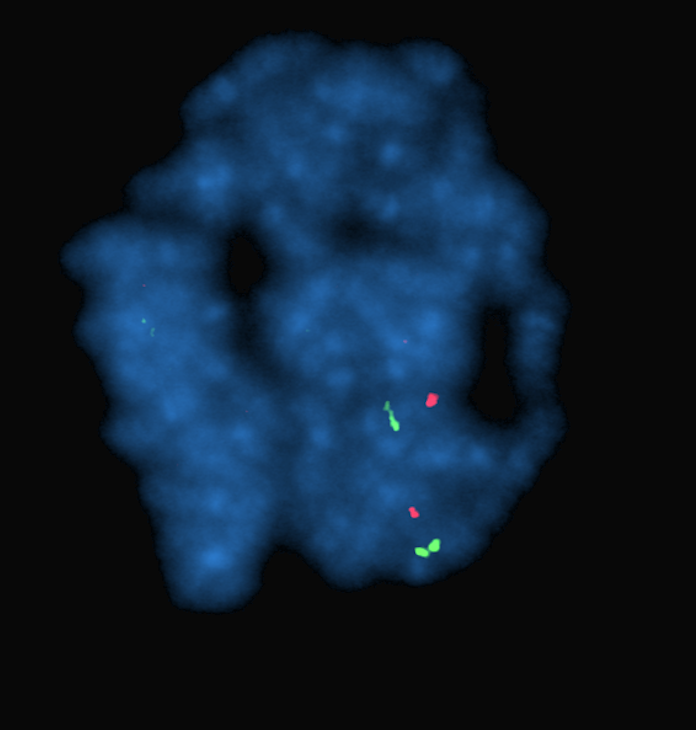


**Supplementary Figure 2.** Image showing probes localised to macaque chromosome 13 (yellow arrows) with red signals obtained on the p arm and green signals on the q arm of chromosome 13.


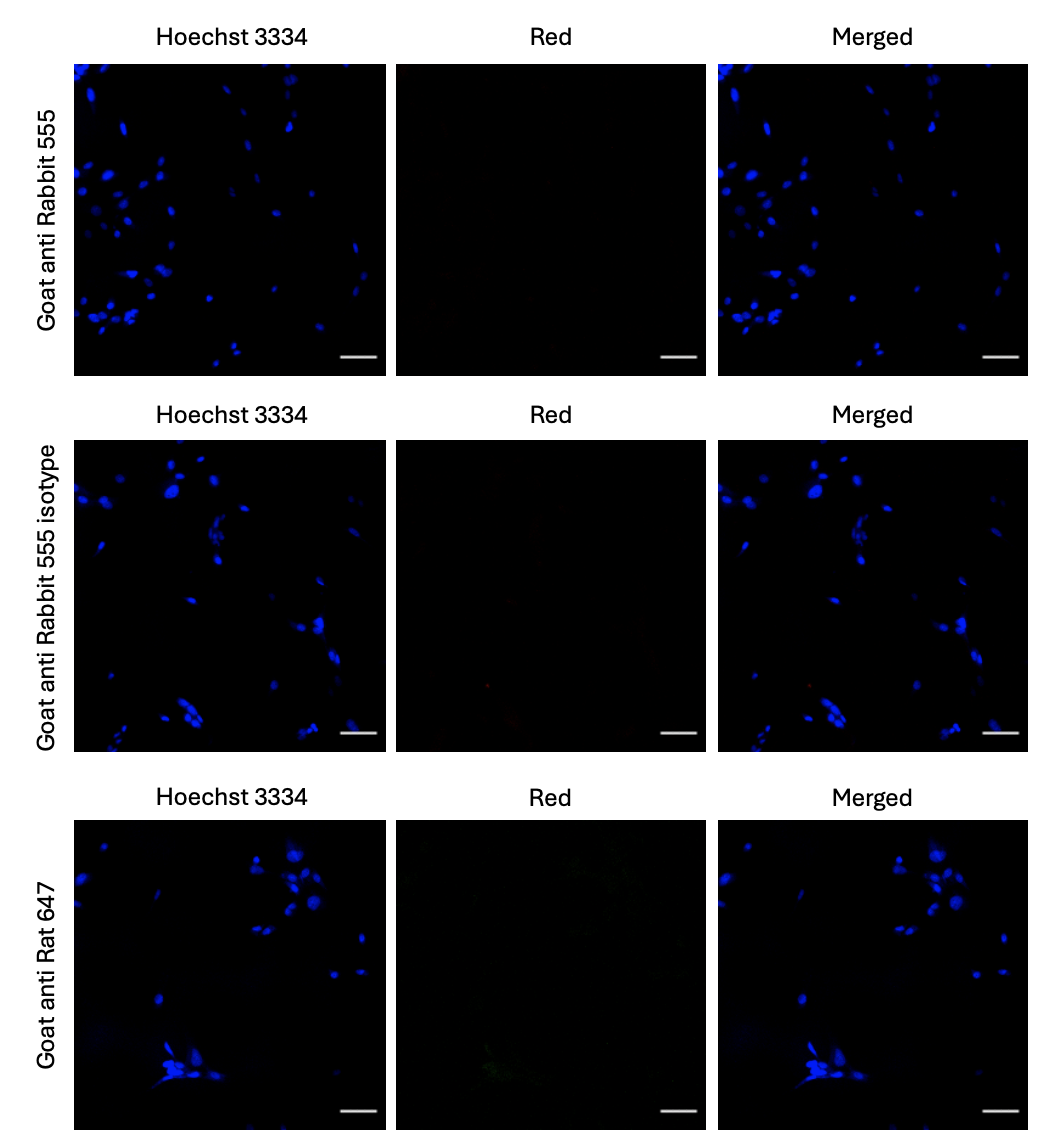


**Supplementary Figure 3**. Negative controls for Müller glia specific markers staining. Confocal images of fixed cells stained with secondary antibody only as controls. (anti-rabbit IgG 555 made in goat, anti-rat IgG 647 made in goat and anti-rabbit IgG 555 isotype control made in goat).


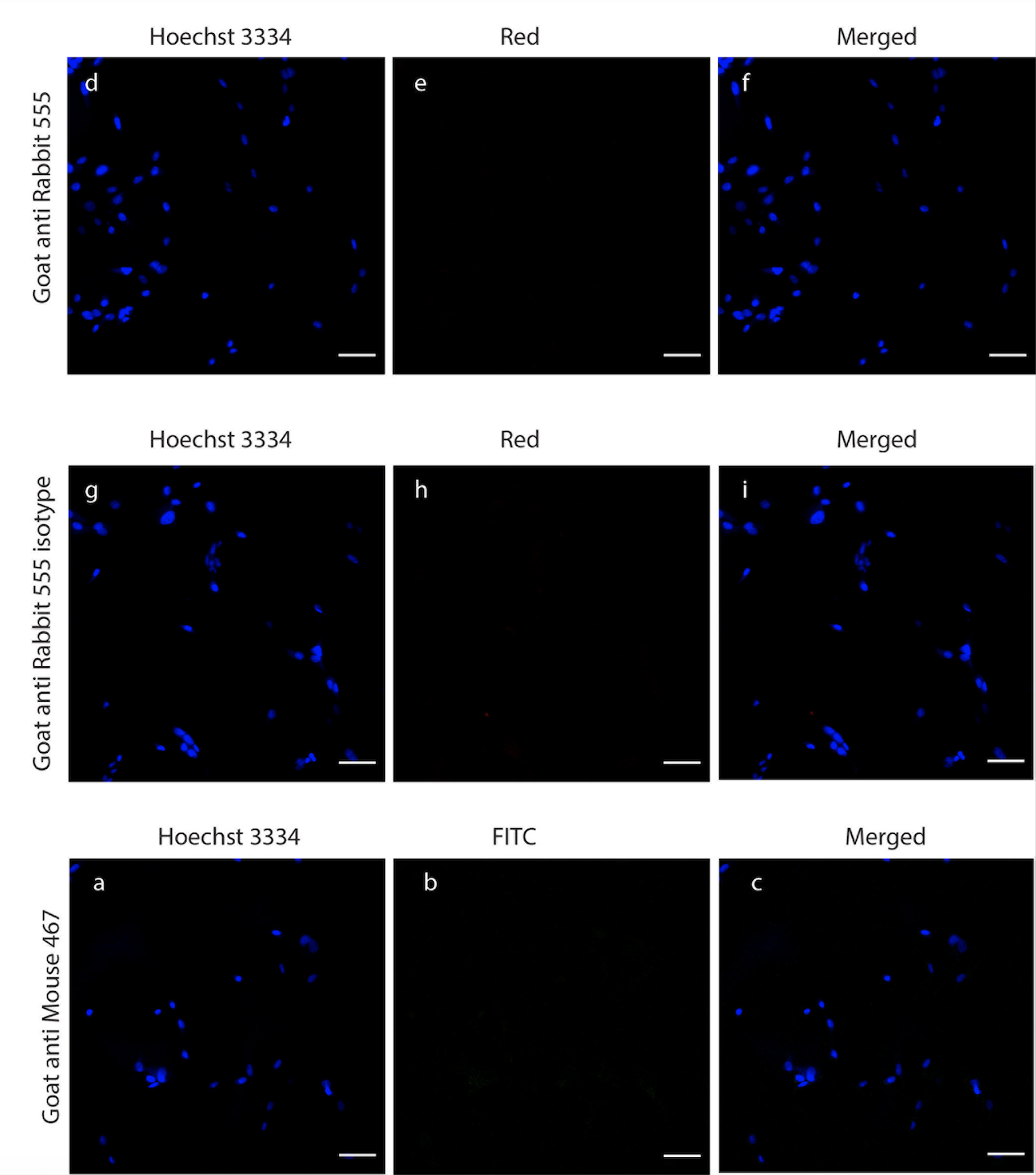


**Supplementary Figure 4**. Negative controls for stem cells specific markers staining. Confocal images of fixed Cells stained with secondary antibody only as controls. (anti-rabbit IgG 555 made in goat, anti-mouse IgG 647 made in goat and anti-rabbit IgG 555 isotype control made in goat).


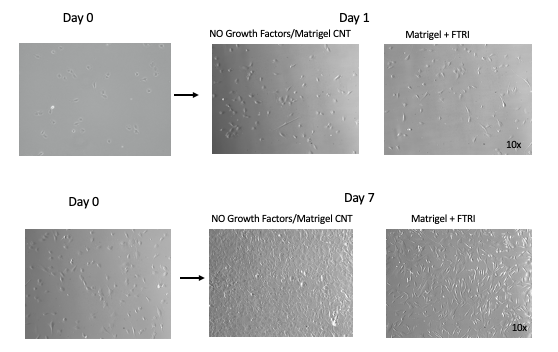


**Supplementary Figure 5**. Shift in Müller glia cells morphology in the presence of FTRI. Sub-confluent monolayer of non-human primate Müller glia cells showing cytoplasmic projections and bipolar shape appearance in the absence of FTRI differentiation medium at Day 1. Density and distribution of cells remarkably reduced with change in morphology 7 days after incubation with the FTRI medium.

A B


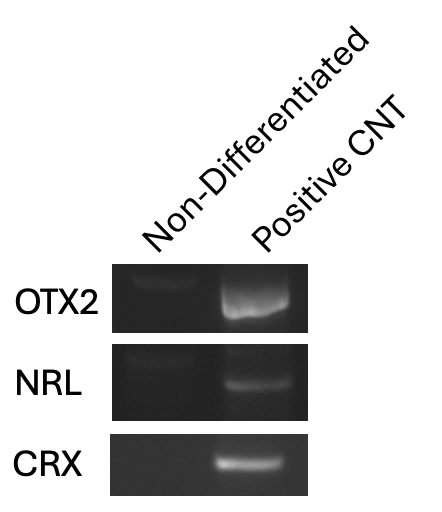

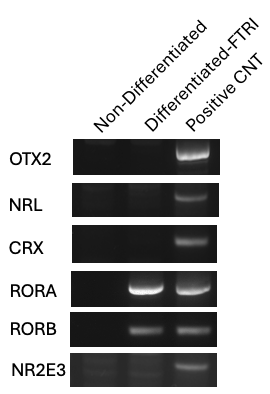


**Supplementary Figure 6**. Expression of early differentiation markers and photoreceptors markers in the NHP spontaneously immortalised Müller cells. (A) Reverse transcription-polymerase reaction (RT-PCR) products showing expression of early differentiation markers homeobox transcription factor (*OTX2),* neural retina leucine zipper (*NRL*) and cone-rod homeobox (*CRX*) by non-differentiated cells. (B) Expression of photoreceptor markers (*OTX2), (NRL), (CRX),* retinoic acid related orphan receptor a and b *(RORA* and *RORB,* respectively*)* and nuclear receptor superfamily 2 group E *(NR2E3)* in non-differentiated and differentiated (FTRI-treated) cells are shown. Primary cells from Rhesus macaque neural retinal culture were used as a positive control to assess the fidelity of PCR primers.
